# 1148. Duration of Antibiotic Therapy in the Treatment of Bacterial Meningitis in Young Infants: A Systematic Review and Narrative Synthesis

**DOI:** 10.1093/ofid/ofab466.1341

**Published:** 2021-12-04

**Authors:** Maite Van Hentenryck, Alan Schroeder, Russell McCulloh, Christopher D Stave, Marie E Wang

**Affiliations:** 1 Stanford University School of Medicine, Palo Alto, California; 2 University of Nebraska Medical Center, Omaha, Nebraska; 3 Lane Medical Library, Stanford University School of Medicine, Palo Alto, California

## Abstract

**Background:**

IDSA recommendations of 14-21 days of parenteral therapy for bacterial meningitis are based predominantly on expert consensus. Parenteral durations consistent with these recommendations are sometimes provided even when meningitis is suspected but not confirmed. We aimed to systematically review the literature on duration of parenteral antibiotic therapy and outcomes in bacterial meningitis in infants < 3 months of age.

**Methods:**

We searched PubMed, Embase, and the Cochrane Central Register of Controlled Trials for publications up until May 11, 2021. Eligible studies were published in English and included infants < 3 months of age with bacterial meningitis for which route and duration of antibiotic therapy and outcomes were reported. We excluded case reports and infants with birth weight < 1500g, major congenital malformations, or neurosurgical conditions. We assessed bias using published tools specific to study type. A meta-analysis was not conducted due to insufficient data on outcomes by duration of therapy. PROSPERO registration: CRD42020201667.

**Results:**

A total of 2195 studies were identified; 280 were selected for full text review and 32 were included for narrative synthesis. There was 1 randomized-controlled trial (RCT), 25 cohort studies, and 6 case series. The RCT found no difference in treatment failure rates between 10 and 14 days of therapy, but only included 2 cerebrospinal fluid (CSF) culture-positive cases. A single cohort study including only CSF culture-negative cases presented outcomes by duration of therapy and concluded that courses >21 days had no impact on prognosis. Twenty-one studies had data on duration of therapy and outcomes by patient, most with small samples (median 4 patients). No conclusions on efficacy of shortened antibiotic courses could be drawn due to small sample sizes and lack of stratification of outcomes by short versus long courses.

**Conclusion:**

Data on parenteral treatment duration in bacterial meningitis in infants < 3 months are primarily observational, and larger studies rarely report outcomes by duration of therapy. Given the associated risks and costs of prolonged parenteral therapy, there is a pressing need for comparative effectiveness research to determine the optimal parenteral treatment duration.

**Disclosures:**

**All Authors**: No reported disclosures

